# PEDOT-Regulated Interfacial Engineering of Sodium Vanadium Oxide Nanostructures for High-Performance Aqueous Zinc-Ion Batteries

**DOI:** 10.3390/nano16120729

**Published:** 2026-06-12

**Authors:** Zeeshan Umar, Jiangfeng Gong, Guangchao Du, Wenyi He, Chunmei Tang, Jingjing Xu, Yuwu Cai, Xinyi Zhao

**Affiliations:** 1 State Key Laboratory of Vanadium and Titanium Resources Comprehensive Utilization, Panzhihua 617000, China; zeeshanzu313@gmail.com (Z.U.);; 2 College of Mechanics and Engineering Science, Hohai University, Nanjing 210098, China; 3 Jiangsu Provincial Institute of Product Quality Supervision and Inspection, Nanjing 210007, China

**Keywords:** aqueous zinc-ion batteries, sodium vanadium oxide, EDOT surface modification, interfacial engineering, cathode materials

## Abstract

Aqueous zinc-ion batteries offer a safe and economical platform for large-scale energy storage, yet vanadium oxide cathodes remain hindered by sluggish Zn^2+^ migration, poor electronic conductivity, and structural degradation during cycling. Herein, a PEDOT regulated interfacial engineering strategy is proposed to construct surface modified sodium vanadium oxide nanostructures with coordinated ion and electron transport. The 1P-NaVO cathode retains the layered framework while introducing a PEDOT-derived surface component that strengthens interfacial charge transfer and preserves accessible Zn^2+^ diffusion pathways, delivering 655 mAh g^−1^ at 0.1 A g^−1^. Kinetic analyses further reveal accelerated charge storage behavior, including an increased pseudocapacitive contribution, a low charge transfer activation energy of 20.6 kJ mol^−1^, and improved Zn^2+^ diffusion, with D_Zn_^2+^ values of approximately 10^−10.8^ to 10^−9.8^ cm^2^ s^−1^. Ex situ XRD and SEM disclose a reversible structural response during Zn^2+^ insertion and extraction, involving interlayer perturbation, local framework relaxation, transient electrolyte-derived surface species, and partial morphology recovery after recharge. These findings show that controlled PEDOT-derived surface regulation promotes efficient coupling between interfacial electron transfer and Zn^2+^ diffusion, offering a practical design principle for durable vanadium-based cathodes in aqueous zinc-ion batteries.

## 1. Introduction

The transition toward low-carbon energy systems has intensified the need for electrochemical storage technologies that are safe, affordable, and durable for stationary and mobile applications. Lithium-ion batteries have achieved remarkable success in portable electronics and electric vehicles, yet their wider use in grid scale storage is still limited by resource scarcity, high cost, and safety concerns. In this context, aqueous zinc-ion batteries (ZIBs) have emerged as promising alternatives, in which metallic zinc acts as the anode and a water-based electrolyte enables ion transport. The natural abundance, low cost, and compatibility of zinc with aqueous electrolytes make these systems attractive for large-scale storage, while the high ionic conductivity of the electrolyte improves operational safety [1]. In addition, the high theoretical capacity of Zn metal, 820 mAh g^−1^, further supports the potential of aqueous ZIBs for practical energy storage [2].

Among the main components of aqueous ZIBs, the cathode largely determines the attainable capacity, rate capability, and cycling stability. Considerable effort has therefore been directed toward developing suitable cathode hosts, including manganese oxides [3,4], Prussian blue analogues [5,6], vanadium-based compounds [7,8,9], and conductive polymers such as polyaniline [10,11]. Vanadium-based oxides are especially attractive because their open structural frameworks and multivalent vanadium redox chemistry provide accessible Zn^2+^ storage sites and enable high reversible capacities [12]. The redox flexibility among V^5+^, V^4+^, and V^3+^ offers abundant charge compensation during Zn^2+^ insertion and extraction, while layered vanadium oxide structures provide channels for ion accommodation. Despite these merits, vanadium oxide cathodes still experience sluggish Zn^2+^ transport and progressive structural degradation during cycling [13]. The strong electrostatic interaction between divalent Zn^2+^ and the vanadium oxygen framework increases the migration barrier, and partial dissolution of vanadium species accelerates capacity decay and weakens rate performance [14]. These issues indicate that efficient ion transport and structural stabilization must be addressed together to advance vanadium-based cathodes for practical aqueous ZIBs.

Interlayer engineering is widely used to mitigate slow Zn^2+^ migration and structural instability in vanadium-based cathodes. In this approach, guest species such as Na^+^ [15], K^+^ [16], Mg^2+^ [17], and Mn related species [18], together with interlayer water molecules, are introduced into the vanadium oxide framework to regulate the interlayer environment. These pre-inserted species enlarge the spacing between adjacent layers, stabilize the host structure, and weaken the strong electrostatic interaction between Zn^2+^ and the vanadium oxygen framework. As a result, the Zn^2+^ migration barrier is reduced, leading to faster diffusion and improved charge discharge kinetics [19]. However, interlayer regulation alone cannot fully overcome the intrinsically poor electronic conductivity of vanadium oxides, which still limits rate performance and active material utilization at high current density. Conductive polymer modification provides a complementary route to address this electronic limitation. Among conducting polymers such as polyaniline, polypyrrole, and poly(3,4 ethylenedioxythiophene), PEDOT is particularly attractive because of its high electronic conductivity, electrochemical stability, and favorable interfacial compatibility with oxide cathodes. When introduced as a surface layer or interfacial conductive network, PEDOT provides additional electron transport pathways, improves electrical contact within the electrode, and limits direct exposure of the vanadium oxide surface to the aqueous electrolyte. This interfacial protection can reduce vanadium dissolution and support more stable cycling during repeated Zn^2+^ insertion and extraction [1,20,21].

Although interlayer engineering and conductive polymer modification have substantially improved vanadium-based cathodes for aqueous ZIBs, previous studies also show that their combined effect depends strongly on the amount and distribution of the polymer component [22]. A well distributed PEDOT layer can enhance electronic transport, strengthen interfacial contact, and help suppress direct dissolution of vanadium species in aqueous electrolytes [23]. However, excessive polymer coverage may form a dense surface barrier that limits electrolyte penetration, extends the Zn^2+^ migration pathway, and restricts access to internal redox active sites. Such behavior can generate a strongly surface-dominated electrochemical response without full utilization of the vanadium oxide framework [1,22]. Conversely, insufficient polymer coverage may provide limited interfacial protection and weak electronic regulation [24]. Thus, the central challenge is to optimize the polymer modification so that it improves charge transfer and interface stability while maintaining open Zn^2+^ diffusion pathways. This balance between electronic conductivity and ion transport motivates the design of PEDOT-regulated NaVO cathodes with controlled surface structure.

In this work, PEDOT-regulated sodium doped vanadium oxide was synthesized through a sonochemical route. This is different from previously reported PEDOT/vanadium oxide systems, which generally rely on hydrothermal/autoclave synthesis, or other relatively high-temperature processing routes [24,25,26]. To clarify the influence of EDOT content on this structural and interfacial balance, three samples denoted as NaVO, 1P-NaVO, and 5P-NaVO were prepared by changing the EDOT amount during synthesis. Among them, 1P-NaVO shows the most favorable electrochemical performance, demonstrating that moderate PEDOT-derived surface modification is more effective than excessive polymer loading. Structural and electrochemical analyses reveal that the hydrated Na-containing vanadium oxide framework provides accessible pathways for Zn^2+^ storage, while the PEDOT-derived surface layer improves electronic contact and interfacial kinetics. The 1P-NaVO electrode with moderate EDOT exhibits an enhanced capacitive contribution, low activation energy, improved Zn^2+^ diffusion behavior, and partial structural recovery during discharge and charge. These results indicate that the improved behavior arises from improved electronic transport while preserving Zn^2+^ diffusion pathways, offering a practical design strategy for durable vanadium-based cathodes in aqueous ZIBs.

## 2. Materials and Methods

### 2.1. Materials

All reagents used in this work were of analytical grade and used without further purification. Commercial V_2_O_5_ powder (99%) was purchased from RHAWN (Shanghai, China). Hydrogen peroxide (H_2_O_2_, 7%), sodium chloride (NaCl, 99%), and 3,4 ethylenedioxythiophene monomer were obtained from KESHI (Shanghai, China).

### 2.2. Synthesis of Na-Doped V_2_O_5_ (NaVO)

NaVO was synthesized through an ultrasonication-assisted chemical process, in which commercial V_2_O_5_ and NaCl were used as the vanadium and sodium sources, respectively. First, 1.0 g of V_2_O_5_ powder was added into 25 mL of H_2_O_2_ solution and stirred magnetically for 35 min. During this step, the V_2_O_5_ gradually dissolved and produced a homogeneous dark brown vanadium precursor solution. Afterward, 275 mL of 0.1 M NaCl aqueous solution was introduced into the precursor solution, adjusting the total reaction volume to 300 mL. The mixed solution was then treated in a water bath ultrasonic cleaner at 60 °C for 6 h, with the instrument operating at 100 W and 80 kHz. As the ultrasonication proceeded, the color of the precursor solution changed from dark brown to red, indicating the formation of sodium-incorporated vanadium oxide species. The resulting red suspension was named Suspension A. Finally, the solid product was recovered by vacuum filtration and dried at 60 °C for 12 h. The product obtained without the addition of EDOT was labeled as NaVO.

### 2.3. Synthesis of PEDOT-Regulated NaVO Composites

PEDOT-regulated NaVO composites were prepared using Suspension A as the precursor suspension. Before EDOT addition, Suspension A was transferred to an ice water bath and stirred for 20 min. EDOT monomer was then added dropwise under continuous stirring to avoid local aggregation. Two EDOT volumes, 1 mL and 5 mL, were used to control the degree of surface modification. After complete addition, the mixtures were stirred for another 30 min and then maintained in an ice water bath for 24 h to promote uniform EDOT-derived surface modification on the NaVO particles. No additional oxidizing agent was introduced. EDOT polymerization was initiated in situ by the V^5+^-containing vanadium species present in Suspension A. The resulting products were washed thoroughly with deionized water and anhydrous ethanol, collected by vacuum filtration, and dried under the same conditions. The samples prepared using 1 mL and 5 mL EDOT were denoted as 1P-NaVO and 5P-NaVO, respectively.

### 2.4. Material Characterizations

The phase structure and crystallographic features of the synthesized samples were investigated by X-ray diffraction using a Rigaku SmartLab SE diffractometer (Rigaku Corporation, Tokyo, Japan) at a scanning rate of 4° min^−1^. Surface morphology and microstructural characteristics were examined by field emission scanning electron microscopy (MIRA4, TESCAN, Brno, Czech Republic) and transmission electron microscopy (Talos F200X, Thermo Fisher Scientific, Waltham, MA, USA), respectively. Energy dispersive X-ray spectroscopy was used to analyze elemental distribution and composition. Fourier transform infrared spectroscopy was performed to identify the chemical bonding environment and structural characteristics of NaVO, 1P-NaVO, and 5P-NaVO. Thermogravimetric analysis was conducted to evaluate water content and estimate the PEDOT-derived organic fraction in the composites. X-ray photoelectron spectroscopy was employed to examine the surface elemental composition and chemical valence states of the prepared samples.

### 2.5. Electrochemical Measurements

The electrochemical properties of the prepared cathodes were examined in CR2032 coin cells. For cell assembly, NaVO, 1P-NaVO, or 5P-NaVO was used as the cathode material, while Zn foil was used as both the counter and reference electrode. Whatman filter paper and 2 mol L^−1^ Zn(CF_3_SO_3_)_2_ aqueous solution was employed as the separator and electrolyte, respectively. The working electrodes were fabricated by preparing a slurry containing active material, carbon black, and polyvinylidene fluoride at a mass ratio of 7:2:1. The electrode constituents were dispersed in 9 mL of N methyl 2 pyrrolidone to obtain a homogeneous slurry, which was subsequently coated onto stainless steel foil. The coated electrodes were dried under vacuum at 60 °C for 12 h, and the final active material loading was maintained between 1.2 and 1.5 mg cm^−2^, corresponding to an average film thickness of ~35 μm. During cell assembly, 70 μL of the electrolyte was added to each coin cell, which was subsequently sealed using a coin cell crimping machine under a hydraulic force of ~800 kg. Galvanostatic charge–discharge measurements and galvanostatic intermittent titration technique analysis were conducted using a Neware CT4008T battery testing system (NEWARE, Shenzhen, China). Cyclic voltammetry and electrochemical impedance spectroscopy were recorded on a CHI660E electrochemical workstation (CH Instruments, Inc., Austin, TX, USA). Details can be found in the Supporting Information.

## 3. Results

**Figure 1:**
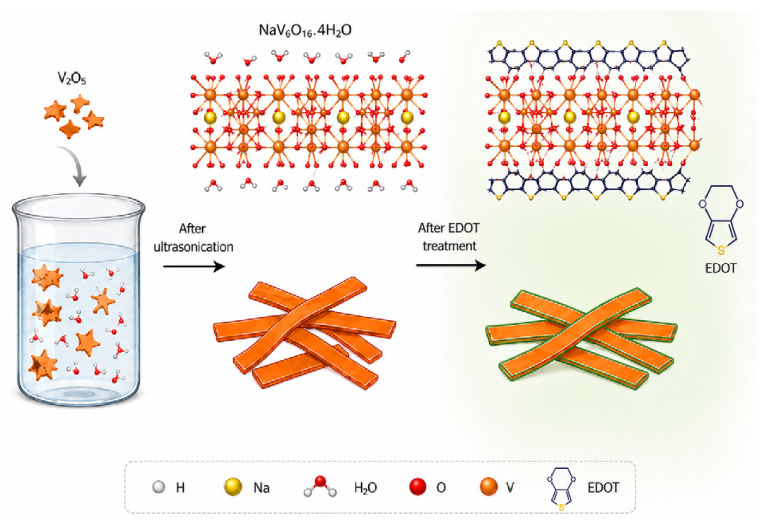
Schematic diagram of the synthesis route for PEDOT-regulated NaVO composites.

The synthesis route for the PEDOT-regulated NaVO composites is shown in Figure 1. First, commercial V_2_O_5_ was activated in H_2_O_2_ to form a soluble vanadium precursor. During this step, H_2_O_2_ interacts with vanadium centers and converts the poorly soluble V_2_O_5_ into reactive peroxovanadate species [27]. The gas release observed during stirring indicates the decomposition of peroxide-containing intermediates and the gradual dissolution of V_2_O_5_. After a homogeneous precursor solution was obtained, NaCl was added as the Na^+^ source. Subsequent ultrasonication improved local mixing and mass transport, allowing Na^+^ to interact effectively with vanadium oxygen species and promoting the formation of the Na containing hydrated vanadium oxide framework. Meanwhile, Na^+^ helped balance charge and stabilize the interlayer structure of NaVO. Overall, Figure 1 summarizes a stepwise synthesis process involving peroxide-assisted V_2_O_5_ activation, Na^+^-directed NaVO formation, and PEDOT-mediated surface modification.

**Figure 2:**
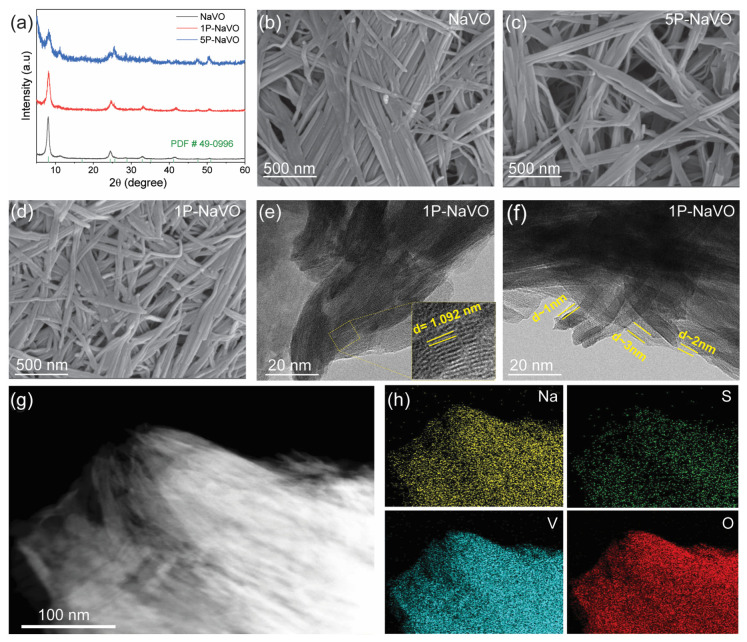
(**a**) XRD patterns of NaVO, 1P-NaVO, and 5P-NaVO. SEM images of (**b**) NaVO, (**c**) 5P-NaVO, and (**d**) 1P-NaVO. (**e**,**f**) TEM images of 1P-NaVO, (**g**) TEM-EDS dark-field image of 1P-NaVO and (**h**) corresponding elemental mappings of Na, S, V, and O.

X-ray diffraction was first used to examine the phase structure of NaVO, 1P-NaVO, and 5P-NaVO. As shown in Figure 2a, the main diffraction peaks of all three samples match layered NaV_6_O_16_·4H_2_O (PDF No. 49-0996), confirming the successful formation of the hydrated sodium vanadium oxide phase. The characteristic low-angle reflection at about 2θ = 8.20° is retained after EDOT treatment, which indicates that the layered framework remains preserved in both modified samples [28]. Notably, this reflection does not show an obvious shift for 1P-NaVO or 5P-NaVO, suggesting that PEDOT modification does not significantly expand the interlayer spacing. This observation implies that PEDOT is mainly located on the external surface of the NaVO nanobelts rather than inserted into the interlayer galleries [29]. Compared with pristine NaVO, 1P-NaVO and 5P-NaVO display lower diffraction intensity and slightly broadened features, indicating reduced crystallinity or partial suppression of long-range structural order after PEDOT formation. The weak reflection near 11° may be associated with changes in hydration state or interlayer water content. Therefore, XRD confirms that PEDOT modification preserves the primary NaVO phase while moderately affecting structural ordering within the layered host.

The morphology and microstructure of NaVO, 1P-NaVO, and 5P-NaVO were further examined by SEM and TEM. As shown in Figure 2b and Figure S1a, pristine NaVO consists of densely interconnected nanobelts with relatively smooth surfaces and clear edges. After EDOT treatment, the belt-like morphology is largely retained in both 5P-NaVO (Figure 2c and Figure S1b) and 1P-NaVO (Figure 2d and Figure S1c,d), indicating that the modification process does not destroy the primary one-dimensional layered morphology. Compared with NaVO, the modified samples show slightly rougher surfaces and less sharply defined edges, which is consistent with the introduction of a thin surface layer. The nanobelt network also appears more open after EDOT treatment, suggesting that the PEDOT component may reduce compact stacking between adjacent belts.

The local structure of 1P-NaVO was analyzed in more detail by TEM. As shown in Figure 2e,f 1P-NaVO maintains the nanobelt morphology, while a clear contrast difference appears between the crystalline inner region and the outer surface. The external region is lighter and more diffuse, without distinct lattice fringes, suggesting the presence of an amorphous coating. The measured lattice spacing of 1.092 nm can be assigned to the (100) plane of layered sodium vanadium oxide [30], which is consistent with the low-angle XRD reflection discussed above. Importantly, this spacing supports preservation of the layered framework rather than a clear PEDOT-induced expansion of the interlayer region. The amorphous surface layer shows slight thickness variation across different areas, with a local thickness estimated at approximately 1 nm to 3 nm. This result supports the formation of a thin PEDOT layer on NaVO nanobelts without major reconstruction of the bulk lattice. Elemental mapping in Figure 2g,h further shows uniform distributions of Na, V, and O, while the presence of S confirms successful introduction of PEDOT on the NaVO surface. These structural observations indicate that EDOT modification maintains the layered NaVO phase and nanobelt architecture while introducing a thin amorphous PEDOT-rich surface layer, which provides the structural basis for later interfacial kinetic improvement.

**Figure 3:**
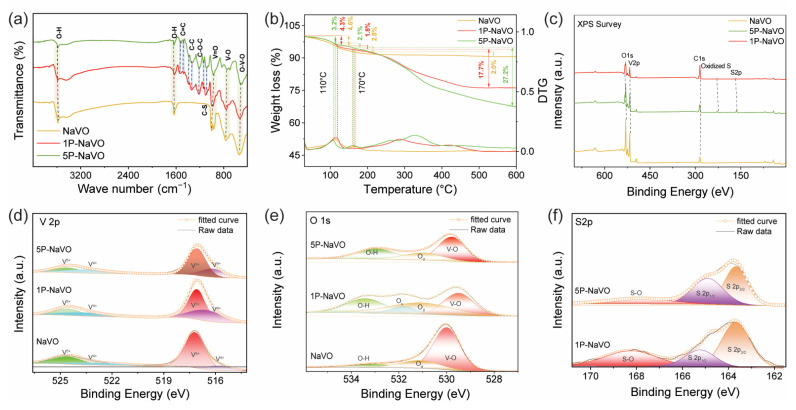
(**a**) FTIR spectra of NaVO, 1P-NaVO, and 5P-NaVO. (**b**) TGA curves. XPS spectra, including (**c**) survey spectra, (**d**) V 2p, (**e**) O 1s, and (**f**) S 2p.

Fourier transform infrared spectroscopy was conducted to clarify the bonding configurations and chemical functionalities of NaVO, 1P-NaVO, and 5P-NaVO. As shown in Figure 3a, pristine NaVO exhibits characteristic vanadate-related bands at approximately 530, 760, and 994 cm^−1^, which can be assigned to O-V-O bending, V-O stretching, and terminal V=O stretching vibrations, respectively. The band near 1634 cm^−1^ is associated with the bending vibration of O-H groups from adsorbed or structural water. After EDOT treatment, these vanadate-related vibrations remain visible in 1P-NaVO and 5P-NaVO, indicating that the main vanadium oxide bonding framework is largely retained. Meanwhile, additional absorption bands appear in the modified samples, verifying the incorporation of PEDOT. The bands at 1084 and 1137 cm^−1^ correspond to C-O and C-S stretching vibrations, respectively [31], while the signal at 1220 cm^−1^ is attributed to C-O-C stretching. The peaks at 1332 and 1386 cm^−1^ are related to C-C vibrational modes, and the band at 1520 cm^−1^ arises from C=C stretching of the conjugated PEDOT backbone [28,29]. The weak band near 3590 cm^−1^ can be assigned to free or weakly hydrogen bonded O-H groups on the sample surface [32]. The PEDOT-related signals become stronger from 1P-NaVO to 5P-NaVO, indicating increased polymer content or more extensive surface coverage with higher EDOT dosage. Thus, FTIR confirms PEDOT modification while showing that the main vanadate bonding environment remains intact.

Thermogravimetric analysis provides further evidence of changes in hydration behavior and polymer content. As shown in Figure 3b, NaVO, 1P-NaVO, and 5P-NaVO display stepwise thermal decomposition. The initial mass loss below approximately 120 °C is assigned to physically adsorbed water and decreases from 4.6% for NaVO to 4.3% and 3.2% for 1P-NaVO and 5P-NaVO, respectively. This decline suggests that PEDOT modification lowers the moisture affinity of the NaVO surface, likely because the polymer layer partially shields the hydrated oxide surface from ambient water [20,33]. The second loss near 170 °C, mainly related to more strongly bound or interlayer water, with possible contribution from residual organic species, is 2.8% for NaVO, 1.8% for 1P-NaVO, and 2.1% for 5P-NaVO. The lower value for 1P-NaVO suggests fewer loosely retained water or labile species, whereas the higher loss in 5P-NaVO is consistent with a larger amount of polymer related components. At higher temperature, the modified samples show pronounced mass losses of 15.70 wt% for 1P-NaVO and 25.2 wt% for 5P-NaVO between approximately 170 and 600 °C, mainly arising from PEDOT decomposition [33,34]. These values were obtained after subtracting the mass loss of pristine NaVO over the same temperature range, allowing a more accurate estimation of the polymer-related contribution. The larger loss of 5P-NaVO confirms its higher PEDOT content, corresponding approximately to the nominal compositions of NaV_6_O_16_·2H_2_O·0.8EDOT for 1P-NaVO and NaV_6_O_16_·2H_2_O·1.5EDOT for 5P-NaVO. These results show that EDOT treatment changes the hydration and surface chemistry of NaVO while introducing a measurable polymer fraction.

X-ray photoelectron spectroscopy was used to investigate the surface composition and valence states of NaVO, 1P-NaVO, and 5P-NaVO. As shown in Figure 3c, the survey spectrum of pristine NaVO mainly contains V 2p and O 1s signals, together with a C 1s peak near 284 eV that is commonly associated with adventitious carbon. After EDOT treatment, sulfur-related signals appear at approximately 226 eV and 162 eV, corresponding to S 2s [35] and S 2p, respectively, confirming the successful introduction of PEDOT [36,37]. These signals are stronger in 5P-NaVO than in 1P-NaVO, which agrees with the larger amount of EDOT. Therefore, the survey spectra verify PEDOT incorporation and support tunable polymer loading in the NaVO composites. High-resolution spectra further clarify the local surface chemistry. As shown in Figure 3d, the V 2p spectra can be fitted into two spin orbit doublets corresponding to V^4+^ at 516.0 and 523.4 eV and V^5+^ at 517.4 and 524.7 eV [38,39]. Compared with NaVO, the PEDOT-regulated samples show a higher relative contribution of V^4+^, indicating partial reduction of V^5+^ during EDOT polymerization. This change is likely related to redox interaction between EDOT and the vanadium oxide surface during oxidative polymerization [20]. The O 1s spectra in Figure 3e can be divided into lattice V−O species at 530.2 eV, defect-related oxygen species at 531.1 eV, hybrid surface-organic oxygen at 531.7 eV (O_s_) [40], and a higher binding energy component near 533.0 eV associated with adsorbed water and oxygen-containing surface groups (O-H) [41,42]. The relative defect oxygen content increases from 19.07% for NaVO to 26.23% for 1P-NaVO and then decreases to 21.43% for 5P-NaVO, suggesting that moderate EDOT modification generates the most evident change in the surface oxygen environment. The 531.7 eV peak is assigned to a combined surface- and organic-oxygen-related signal, encapsulating overlapping contributions from surface hydroxyls and polymer-associated functional groups (such as C=O, O-C, and S=O configurations) [43,44]. This result indicates that 1P-NaVO may provide more electrochemically active surface sites and improved interfacial charge transfer. The S 2p spectra of 1P-NaVO and 5P-NaVO in Figure 3f show peaks at 163.7, 164.8, and 168.2 eV, corresponding to S 2p_3/2_, S 2p_1/2_, and oxidized S-O bonding, respectively. The C 1s spectrum in Figure S2a contains peaks at 284.8, 286.3, and 288.0 eV, which can be assigned to C-C or C=C bonding, C-S or C-O bonding, and oxygenated carbon species from the PEDOT component [20,37,45,46]. This surface chemical modification suggests that EDOT treatment not only introduces PEDOT-related species but also adjusts the valence and defect environment of NaVO, which is expected to support improved electrochemical kinetics.

### Electrochemical Performance

**Figure 4:**
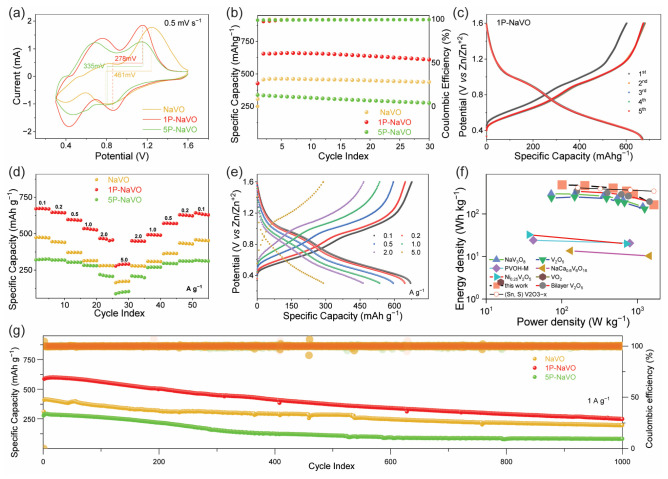
(**a**) CV curves of NaVO, 1P-NaVO, and 5P-NaVO at 0.5 mV s^−1^. (**b**) Cycling performance at 0.1 A g^−1^. (**c**) First five charge–discharge curves of 1P-NaVO at 0.1 A g^−1^. (**d**) Rate performance comparison. (**e**) Charge–discharge profiles of 1P-NaVO at different current densities. (**f**) Ragone plot. (**g**) Long-term cycling performance of the three electrodes and corresponding Coulombic efficiency of the three electrodes at 1 A g^−1^.

The electrochemical behavior of NaVO, 1P-NaVO, and 5P-NaVO was first evaluated by cyclic voltammetry in CR2032 coin cells using Zn foil as the anode and 2 mol L^−1^ Zn(CF_3_SO_3_)_2_ as the electrolyte. As shown in Figure 4a, the third-cycle CV curves recorded at 0.5 mV s^−1^ display two pairs of distinct redox peaks within 0.3 to 1.6 V, indicating a multistep and reversible charge storage process. These redox features are associated with stepwise H^+^ and Zn^2+^ insertion and extraction in the layered vanadium oxide host, accompanied by reversible vanadium redox reactions [47]. Although all electrodes show similar profiles, 1P-NaVO exhibits the largest integrated CV area and the highest redox peak current, demonstrating enhanced electrochemical activity and a larger charge storage contribution. More importantly, the peak separation of 1P-NaVO is 278 mV, smaller than that of 5P-NaVO (335 mV) and NaVO (461 mV), indicating lower electrochemical polarization and faster redox kinetics after moderate PEDOT modification. In contrast, the broader peak separation and weaker current response of 5P-NaVO suggest that excessive PEDOT loading may restrict ion accessibility or partially shield active sites. These results show that 1P-NaVO provides the most favorable balance between electronic enhancement and Zn^2+^ transport accessibility.

The cycling behavior at low current density further supports this conclusion. As shown in Figure 4b, 1P-NaVO delivers a high discharge capacity of 655.86 mAh g^−1^ at 0.1 A g^−1^ and retains 610.36 mAh g^−1^ after 30 cycles, remaining clearly higher than NaVO and 5P-NaVO. This enhanced capacity is consistent with the larger CV response and reduced polarization discussed above, indicating that moderate PEDOT modification improves electrochemical utilization of the layered NaVO host. The initial five charge–discharge profiles of 1P-NaVO in Figure 4c show similar curve shapes with well-defined voltage plateaus, suggesting a reversible multistep Zn^2+^ and H^+^ storage process during the early cycles. The rate performance in Figure 4d further confirms the advantage of 1P-NaVO. The electrode delivers capacities of 672.30, 645.44, 594.99, 529.01, 462.94, and 289.35 mAh g^−1^ at 0.1, 0.2, 0.5, 1.0, 2.0, and 5.0 A g^−1^, respectively. These values exceed those of NaVO, which provides 474.86, 443.52, 371.67, 314.58, 280.37, and 172.45 mAh g^−1^, consistent with the theoretical capacity value, and 5P-NaVO, which delivers 324.07, 319.46, 304.22, 281.17, 214.30, and 101.53 mAh g^−1^ at the same current densities. When the current density returns to 1.0 A g^−1^, 1P-NaVO recovers a capacity of 491 mAh g^−1^, confirming good rate reversibility. The theoretical capacity of the NVO host is ~550 mAh g^−1^. The 1P-NaVO capacity of 672 mAh g^−1^ exceeds the theoretical value due to synergistic contributions from PEDOT pseudocapacitance, interfacial/surface defect storage, and enhanced electronic contact, enabling fuller active-site utilization [48,49]. The corresponding charge discharge profiles in Figure 4e retain distinguishable voltage features at different current densities, further indicating that the electrode with moderate PEDOT maintains active redox behavior under faster cycling conditions.

The practical energy output of 1P-NaVO was compared with reported vanadium-based cathodes, including PVOH-M, NaCa_0.6_V_6_O_16_·3H_2_O, VO_2_, bilayer V_2_O_5_, NaV_3_O_8_, and V_2_O_5_ [15,50,51,52,53]. As shown in the Ragone plot in Figure 4f, 1P-NaVO achieves an energy density of 163.39 Wh kg^−1^ at a power density of 1650.40 W kg^−1^, indicating competitive energy storage performance among aqueous zinc-ion battery cathodes. Long-term cycling at 1.0 A g^−1^ is shown in Figure 4g. 1P-NaVO delivers an initial capacity of 587.31 mAh g^−1^ and retains 248.94 mAh g^−1^ after 1000 cycles, corresponding to a retention of approximately 42.4%. Under the same conditions, NaVO decreases from 410.16 to 198.02 mAh g^−1^, while 5P-NaVO decreases from 290.10 to 82.29 mAh g^−1^. Although 1P-NaVO still undergoes capacity fading during prolonged cycling, it maintains the highest reversible capacity among the three electrodes. From a literature-supported interpretation of layered vanadium oxide electrodes, the gradual decay may arise from structural fatigue of the layered vanadium oxide host, partial vanadium dissolution in aqueous electrolytes, and incomplete suppression of active material loss during repeated ion insertion and extraction. For 5P-NaVO, the poorer cycling behavior is likely caused by excessive PEDOT loading, which can hinder ion diffusion, restrict electrolyte access, and reduce the fraction of active vanadium oxide per unit electrode mass [22]. The distinction between moderate and excessive PEDOT modification was evaluated from the combined TGA, SEM, and electrochemical results, indicating that an appropriate amount of PEDOT modification provides a better balance of capacity retention, rate capability, and long-term stability compared with unmodified NaVO and over-coated PEDOT samples.

**Figure 5:**
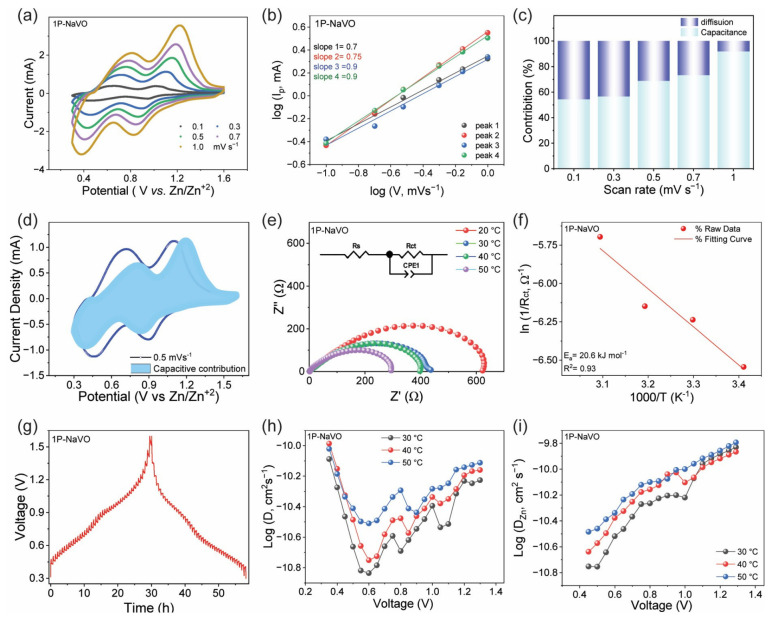
(**a**) CV curves of 1P-NaVO at various scan rates. (**b**) The b values calculated from log v versus log i plots for four redox peaks. (**c**) Capacitive and diffusion-controlled contribution percentages at different scan rates. (**d**) Capacitive contribution at 0.5 mV s^−1^. (**e**) Temperature-dependent Nyquist plots for 1P-NaVO. (**f**) Arrhenius plot with calculated activation energy for the Zn//1P-NaVO cell. (**g**) GITT voltage profile of 1P-NaVO. (**h**) Calculated D_Zn_^2+^ values during discharge at different temperatures. (**i**) Calculated D_Zn_^2+^ values during charge at different temperatures.

A systematic kinetic analysis was performed to clarify the charge storage behavior of NaVO, 1P-NaVO, and 5P-NaVO. Cyclic voltammetry was first recorded at scan rates from 0.1 to 1.0 mV s^−1^. As shown in Figure 5a and Figures S3a and S4a, all electrodes display multiple redox peaks, confirming multistep Zn^2+^ insertion and extraction within the vanadium oxide framework [54]. With increasing scan rate, 1P-NaVO maintains clearer and more defined redox peaks than NaVO and 5P-NaVO, indicating improved electrochemical reversibility and faster reaction kinetics after moderate EDOT modification. This improvement is attributed to enhanced interfacial contact and electronic transport, while the NaVO framework remains accessible for Zn^2+^ diffusion.

To further distinguish the kinetic contribution, the relationship i = av^b^ was applied, where b values close to 0.5 and 1.0 correspond to diffusion-controlled and surface-controlled capacitive processes, respectively [55]. The b values of 1P-NaVO are 0.70, 0.75, 0.90, and 0.90 for the four redox peaks (Figure 5b), revealing a mixed charge storage mechanism with a strong pseudocapacitive contribution [56]. In comparison, NaVO shows lower b values of 0.30, 0.70, 0.80, and 0.60 (Figure S3b), suggesting stronger dependence on diffusion-controlled reactions. Although 5P-NaVO exhibits higher b values of 0.83, 0.89, 0.96, and 0.98 (Figure S4b), its broader and less-resolved CV peaks at higher scan rates indicate that excessive EDOT modification promotes surface-dominated charge storage, without efficient utilization of the inner NaVO framework. This behavior is likely related to a thicker PEDOT-derived layer, which can partially cover electroactive sites and increase the transport distance for Zn^2+^ migration from the electrolyte to the vanadium oxide host.

The capacitive and diffusion-controlled contributions were then quantified using i(V) = k_1_v + k_2_v^1/2^, where k_1_v represents the surface-controlled capacitive current and k_2_v^1/2^ corresponds to the diffusion-controlled current [50]. As shown in Figure 5c and Figures S3c and S4c, the capacitive contribution increases with scan rate for all electrodes, indicating that surface-controlled kinetics become more prominent at higher polarization rates. For NaVO, the capacitive contribution increases from 50.14% at 0.1 mV s^−1^ to 77.26% at 1.0 mV s^−1^. After moderate EDOT modification, 1P-NaVO shows capacitive contributions of 54.4%, 56.5%, 68.8%, 73.3%, and 91.9% from 0.1 to 1.0 mV s^−1^, confirming enhanced surface reaction kinetics compared with pristine NaVO. In contrast, 5P-NaVO exhibits the highest capacitive fractions of 59.32%, 59.82%, 75.55%, 81.96%, and 93.25%, suggesting a stronger surface-dominated response after excessive EDOT addition. At 0.5 mV s^−1^, the capacitive contribution follows the order NaVO (70.46%) < 1P-NaVO (76.8%) < 5P-NaVO (81.96%), as shown in Figure 5d and Figures S3d and S4d. This trend agrees with the b value analysis and confirms that EDOT modification gradually shifts the storage mechanism from diffusion-influenced behavior toward faster surface-controlled kinetics.

Temperature-dependent EIS was further performed to evaluate interfacial charge transfer kinetics. As shown in Figure 5e and Figure S5, the charge transfer resistance decreases gradually with increasing temperature for all electrodes, demonstrating a thermally activated interfacial reaction process. The corresponding activation energy was calculated from the Arrhenius relationship between charge transfer resistance and temperature [50]. As shown in Figure 5f, 1P-NaVO delivers a low activation energy of 20.6 kJ mol^−1^, indicating a reduced kinetic barrier for interfacial charge transfer and Zn^2+^ transport across the electrode electrolyte interface [57]. In comparison, NaVO shows a slightly higher value of 21.28 kJ mol^−1^ (Figure S5b), consistent with less favorable charge transfer in the bare framework. A different trend appears for 5P-NaVO. Although its apparent charge transfer resistance is lower than that of NaVO, the calculated activation energy increases markedly to 52.37 kJ mol^−1^ (Figure S5d). This result suggests that excessive EDOT modification does not simply improve the overall interfacial kinetics. Instead, a thick PEDOT-derived surface layer may lower apparent electronic resistance while increasing the energetic barrier for Zn^2+^ migration from the electrolyte into the vanadium oxide host. This interpretation agrees with the CV analysis, where 5P-NaVO shows a highly surface-dominated response but less well-resolved redox behavior at higher scan rates [19,47].

The Zn^2+^ diffusion behavior of NaVO, 1P-NaVO, and 5P-NaVO was further evaluated by GITT. The voltage profiles and corresponding D_Zn_^2+^ values provide insight into both apparent diffusion kinetics and accessibility of the NaVO host framework [21]. Pristine NaVO shows D_Zn_^2+^ values mainly in the range of 10^−11^ to 10^−10^ cm^2^ s^−1^, indicating that Zn^2+^ insertion and extraction can proceed within the layered structure but remain limited by relatively slow ion and electron transport. After moderate EDOT modification, 1P-NaVO exhibits higher D_Zn_^2+^ values of approximately 10^−10.8^ to 10^−9.8^ cm^2^ s^−1^, together with a more stable GITT voltage response. This result indicates that the moderate PEDOT-derived surface modification facilitates Zn^2+^ transport while maintaining accessible diffusion pathways within the NaVO framework [24]. In contrast, 5P-NaVO delivers lower D_Zn_^2+^ values, mainly around 10^−11.5^ to 10^−10.8^ cm^2^ s^−1^, and displays more evident polarization in the GITT voltage profile. This behavior suggests that excessive EDOT modification limits effective utilization of the internal NaVO framework, most likely because a thicker surface layer increases resistance for Zn^2+^ migration from the electrolyte to the active vanadium oxide sites [19,21]. Temperature-dependent GITT further shows that D_Zn_^2+^ increases with increasing temperature for all electrodes, confirming the thermally activated nature of Zn^2+^ diffusion. Crucially, the continuous D_Zn_^2+^ curves (Figure 5h,i) reveal a distinct kinetic valley at ~0.6 V during discharge due to phase-boundary resistance and localized electrostatic repulsion, followed by a monotonic increase during charging as the host channels clear out. The kinetic data therefore show that 1P-NaVO avoids the transport limitations associated with excessive coating while still improving pseudocapacitive behavior, interfacial activation, and Zn^2+^ diffusion across the electrode (Table S1).

**Figure 6:**
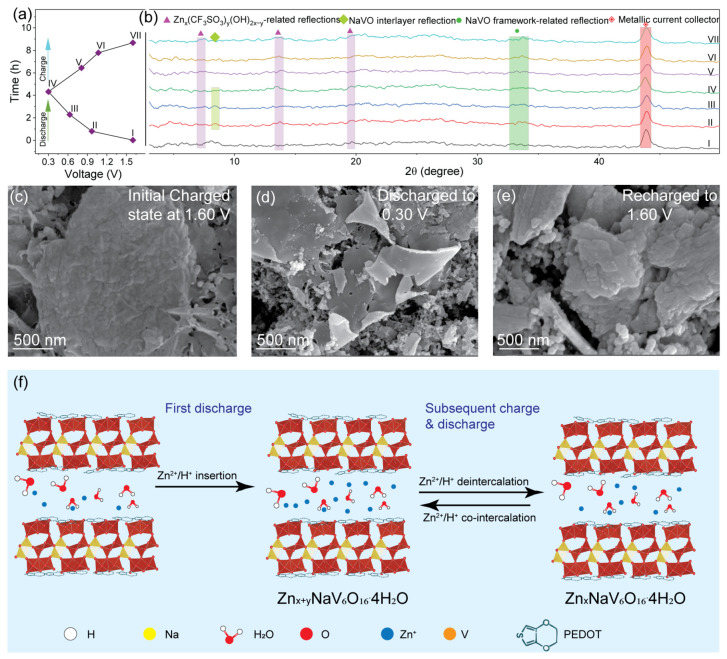
(**a**) Charge–discharge profile with selected states for ex situ analysis. (**b**) Ex situ XRD patterns collected at different charge discharge states. Ex situ SEM images of 1P-NaVO at (**c**) the initial charged state of 1.60 V, (**d**) the discharged state of 0.30 V, and (**e**) the recharged state of 1.60 V. (**f**) Schematic illustration of the proposed Zn^2+^ and H^+^ insertion and extraction mechanism.

The structural response associated with Zn^2+^ storage in 1P-NaVO was examined by ex situ XRD and SEM. As shown in Figure 6a,b (which tracks seven distinct states from I to VII, including intermediate depths), weak reflections appear at approximately 6.7°, 13.9°, and 19.8° during discharge. These peaks can be assigned to Zn_x_(CF_3_SO_3_)_y_(OH)_2x−y_ related surface byproducts formed through the interaction among Zn^2+^, OH^−^ species, and the CF_3_SO_3_^−^ based electrolyte [26,48]. The intensity of these reflections decreases after recharging, indicating that these species are mainly state-dependent products rather than permanent crystalline degradation phases. However, possible gradual accumulation of such byproducts during long-term cycling cannot be completely excluded. The characteristic low-angle reflection of the NaVO host near 8.1° remains visible during the early discharge stage but becomes less distinct at the fully discharged state of 0.30 V. This evolution suggests that Zn^2+^ insertion perturbs interlayer ordering in the hydrated NaVO framework [26,49]. After recharging to 1.60 V, the partial recovery of this reflection indicates Zn^2+^ extraction and retention of the layered host structure. In addition, the broad framework-related feature in the 32 to 34° region shows reversible profile variation during discharge and charge. Because this reflection is broad and weak, exact peak shift analysis is not reliable. Nevertheless, its evolution is consistent with local lattice relaxation of the V-O framework rather than complete structural collapse [22]. Ex situ SEM further supports the structural evolution indicated by XRD. In the initial charged state at 1.60 V, 1P-NaVO displays compact plate-like aggregates with a relatively continuous surface (Figure 6c). After discharge to 0.30 V, the surface becomes rougher and partially fragmented, with granular deposits distributed across the electrode surface (Figure 6d). This morphological change agrees with the appearance of byproduct-related reflections in the discharged XRD pattern. Upon recharging to 1.60 V, a more compact morphology is partly restored, while the main plate-like framework remains recognizable, without severe pulverization (Figure 6e).

The proposed Zn^2+^ storage process of 1P-NaVO is illustrated in Figure 6f. During discharge, Zn^2+^ enters the hydrated NaVO framework, leading to interlayer perturbation and local relaxation of the vanadium oxygen framework. The pre-inserted Na^+^ ions and structural water help preserve the layered environment and reduce the strong interaction between Zn^2+^ and the host lattice. Meanwhile, the higher defect oxygen contribution observed by XPS may provide a more flexible local coordination environment, supporting Zn^2+^ accommodation. The weak Zn_x_(CF_3_SO_3_)_y_(OH)_2x−y_ related reflections detected during discharge indicate the temporary formation of electrolyte-derived surface species. Upon charging, partial recovery of the low-angle NaVO reflection and electrode morphology suggests Zn^2+^ extraction and retention of the main layered structure. Overall, Figure 6 confirms that Zn^2+^ storage in 1P-NaVO involves reversible interlayer perturbation, local framework adjustment, transient electrolyte-derived surface species, and partial morphology recovery, which together help the electrode tolerate discharge-induced structural strain.

## 4. Conclusions

In summary, PEDOT-regulated hydrated sodium vanadium oxide cathodes were synthesized to investigate the effect of surface modification on aqueous zinc-ion battery performance. Structural characterization confirms retention of the layered NaVO framework with surface-integrated organic components. Electrochemical evaluation reveals that 1P-NaVO delivers the best overall performance among the prepared samples, achieving a high discharge capacity of 655 mAh g^−1^ at 0.1 A g^−1^. Kinetic analysis reveals accelerated charge storage via balanced pseudocapacitive and diffusive contributions, a low charge-transfer activation energy of 20.6 kJ mol^−1^, and improved Zn^2+^ diffusion (~10^−10.8^ to 10^−9.8^ cm^2^ s^−1^). Ex situ XRD and SEM confirm that 1P-NaVO undergoes reversible interlayer perturbation, local framework adjustment, the temporary formation of electrolyte-derived surface species, and partial morphology recovery during Zn^2+^ insertion and extraction. These results indicate that the superior behavior of 1P-NaVO arises from balanced regulation of interfacial charge transfer, surface-controlled kinetics, Zn^2+^ diffusion, and structural tolerance. This work highlights that moderate EDOT modification, rather than maximum polymer loading, is more effective for improving hydrated vanadium oxide cathodes and provides a useful design strategy for high performance aqueous ZIBs.

## Data Availability

The original contributions presented in this study are included in the article/Supplementary Materials. Further inquiries can be directed to the corresponding author.

## Supplementary Materials

The following supporting information can be downloaded at: https://www.mdpi.com/article/10.3390/nano16120729/s1, Figure S1: SEM images of NaVO, 5P-NaVO and 1P-NaVO; Figure S2: XPS high resolution of Na 1s and C 1s; Figure S3: CV-related kinetic analysis of NaVO; Figure S4: CV-related electrochemical kinetics of 5P-NaVO; Figure S5: EIS tests results for NaVO and 5P-NaVO at different temperatures; Figure S6: GITT analysis of NaVO and 5P-NaVO. Table S1: Reported D_Zn_^2+^ values of the vanadium-based cathodes. Refs [58,59,60,61] are cited in the supplementary materials.

## References

[1] Shin J., Choi J.W. (2020). Opportunities and Reality of Aqueous Rechargeable Batteries. Adv. Energy Mater..

[2] Wang X., Li Y., Wang S., Zhou F., Das P., Sun C., Zheng S., Wu Z. (2020). 2D Amorphous V_2_O_5_/Graphene Heterostructures for High-Safety Aqueous Zn-Ion Batteries with Unprecedented Capacity and Ultrahigh Rate Capability. Adv. Energy Mater..

[3] Fu Y., Wei Q., Zhang G., Wang X., Zhang J., Hu Y., Wang D., Zuin L., Zhou T., Wu Y. (2018). High-Performance Reversible Aqueous Zn-Ion Battery Based on Porous MnO_x_ Nanorods Coated by MOF-Derived N-Doped Carbon. Adv. Energy Mater..

[4] Sun W., Wang F., Hou S., Yang C., Fan X., Ma Z., Gao T., Han F., Hu R., Zhu M. (2017). Zn/MnO_2_ Battery Chemistry With H^+^ and Zn^2+^ Coinsertion. J. Am. Chem. Soc..

[5] Zeng Y., Lu X.F., Zhang S.L., Luan D., Li S., Lou X.W.D. (2021). Construction of Co–Mn Prussian Blue Analog Hollow Spheres for Efficient Aqueous Zn-ion Batteries. Angew. Chem. Int. Ed..

[6] Li M., Maisuradze M., Sciacca R., Hasa I., Giorgetti M. (2023). A Structural Perspective on Prussian Blue Analogues for Aqueous Zinc-Ion Batteries. Batter. Supercaps.

[7] Yi T.-F., Qiu L., Qu J.-P., Liu H., Zhang J.-H., Zhu Y.-R. (2021). Towards High-Performance Cathodes: Design and Energy Storage Mechanism of Vanadium Oxides-Based Materials for Aqueous Zn-Ion Batteries. Coord. Chem. Rev..

[8] Li Y., Zhang D., Huang S., Yang H.Y. (2021). Guest-Species-Incorporation in Manganese/Vanadium-Based Oxides: Towards High Performance Aqueous Zinc-Ion Batteries. Nano Energy.

[9] Fei B., Liu Z., Fu J., Guo X., Li K., Zhang C., Yang X., Cai D., Liu J., Zhan H. (2023). In Situ Induced Core–Shell Carbon-Encapsulated Amorphous Vanadium Oxide for Ultra-Long Cycle Life Aqueous Zinc-Ion Batteries. Adv. Funct. Mater..

[10] Rahman S.U., Dan X., Farooq S., Sajid M., Tao F.-Y., Rafiq M., Ali U., Xu W.-J., Liu C., Zhang J. (2026). Interfacial Binary Doping Strategy to Achieve High Capacity and Cyclic Stability in Polyaniline Cathodes for Aqueous Zinc Ion Batteries. Colloids Surf. A Physicochem. Eng. Asp..

[11] Gong J., Li H., Zhang K., Zhang Z., Cao J., Shao Z., Tang C., Fu S., Wang Q., Wu X. (2022). Zinc-Ion Storage Mechanism of Polyaniline for Rechargeable Aqueous Zinc-Ion Batteries. Nanomaterials.

[12] Biemolt J., Jungbacker P., Van Teijlingen T., Yan N., Rothenberg G. (2020). Beyond Lithium-Based Batteries. Materials.

[13] Gou W., Kong X., Wang Y., Ai Y., Liang S., Pan A., Cao G. (2019). Yolk-Shell Structured V_2_O_3_ Microspheres Wrapped in N, S Co-Doped Carbon as Pea-Pod Nanofibers for High-Capacity Lithium-ion Batteries. Chem. Eng. J..

[14] Volkov F.S., Tolstopyatova E.G., Eliseeva S.N., Fu L., Kondratiev V.V. (2026). Cobalt-Preintercalated Vanadium Oxide and Its Composite with PEDOT as Cathodes for Aqueous Zinc-Ion Batteries. Mater. Lett..

[15] Lin H., Gong J., Guan Y., Shao Z., Tang C., Yao H., He W., Du G. (2025). Sonochemical Synthesis of Bilayer V_2_O_5_ for Zinc-Ion Batteries. Chem. Eng. J..

[16] Liu M., Li Z., Zhang Y. (2023). K-Doped V_2_O_5_ Derived from V-MOF Precursor as High-Performance Cathode for Aqueous Zinc-Ion Batteries. J. Electroanal. Chem..

[17] Luo S., Cui J., Liang S., Guo Y., Yuan B., Xu L., Zheng R., Li J., Yang W., Chen M. (2024). Graphene-Supported Mg^2+^ Intercalated V2 O5 Nanoribbons as Cathode for Aqueous Zinc-Ion Batteries. ACS Appl. Nano Mater..

[18] Liu H., Wang N., Hu L., Sun M., Li Z., Jia C. (2023). Constructing Graphene Conductive Networks in Manganese Vanadate as High-Performance Cathode for Aqueous Zinc-Ion Batteries. Electrochim. Acta.

[19] Kundu D., Adams B.D., Duffort V., Vajargah S.H., Nazar L.F. (2016). A High-Capacity and Long-Life Aqueous Rechargeable Zinc Battery Using a Metal Oxide Intercalation Cathode. Nat. Energy.

[20] Ren Z., Zhang Y., Lv T., Tan X., Jiang H., Zhou Z., Meng C. (2025). Poly(3,4-Ethylenedioxithiophene) Coated on Vanadium Oxide Hydration Nanobelts Enhancing Ammonium-Ion Storage for Hybrid Supercapacitors. J. Colloid Interface Sci..

[21] Jiang X., Wang T., Ji M., Ji D., Deng S., Gao G., Shen J., Wu G. (2025). Enhancement of De-Solvation Kinetics on V_5_O_12_·6H_2_O Cathode Through a Bi-Functional Modification Layer for Low-Temperature Zinc-Ion Batteries. Adv. Funct. Mater..

[22] Wu R., Zhang Y., Wei M., Wang Y., Li X., Zhang J., Hu K., Qu S., Liu C., Jia D. (2026). Scalable PEDOT Coating Strategy Unlocking High-Rate and Durable Performance of Vanadium Oxide Cathodes. J. Alloys Compd..

[23] Tan S., Sang Z., Yi Z., Guo J., Zhang X., Li P., Si W., Liang J., Hou F. (2023). Conductive Coating, Cation-intercalation, and Oxygen Vacancies Co-modified Vanadium Oxides as High-rate and Stable Cathodes for Aqueous Zinc-ion Batteries. EcoMat.

[24] Liu A., Wang W., Zhang J., Mo F. (2024). Conductive Polymer-Modified Sodium Ion Intercalation in Vanadium Pentoxide for High Performance Zinc-Based Batteries. J. Electroanal. Chem..

[25] Chen C., Hou B., Cheng T., Wu F., Hu Y., Dai Y., Zhang X., Tian Y., Zhao X., Wang L. (2025). Sodium-Intercalated Vanadium Oxide Coated on Carbon Cloth for Electrode Materials in High-Performance Aqueous Zinc-Ion Batteries. Molecules.

[26] So Y., Seo H., Lee S.H., Lee E., Lee J., Kang J., Kim Y.Y., Kim B.-H., Mhin S. (2025). Enhanced Electrochemical Performance of Aqueous Zn-Ion Batteries Based on Na_2_V_6_O_16_·2H_2_O Cathodes: Insights from DFT and Synchrotron X-Ray Analysis. J. Mater. Chem. A.

[27] Fontenot C.J., Wiench J.W., Pruski M., Schrader G.L. (2000). Vanadia Gel Synthesis via Peroxovanadate Precursors. 1. In Situ Laser Raman and 51 V NMR Characterization of the Gelation Process. J. Phys. Chem. B.

[28] Xu D., Wang H., Li F., Guan Z., Wang R., He B., Gong Y., Hu X. (2019). Conformal Conducting Polymer Shells on V_2_O_5_ Nanosheet Arrays as a High-Rate and Stable Zinc-Ion Battery Cathode. Adv. Mater. Inter..

[29] Zhu Y., Cao K., Chen F., Dong J., Ren N., Chen C. (2023). Fine Valence Regulation of Hydrated Vanadium Oxide as a Novel Cathode for Stable Potassium-Ion Storage. Chem. Commun..

[30] Shi L., Jia C., Zhang X., Liang S., Fu Y., Chen Z., Liu X., Wan F., Zhang L. (2022). Engineering the Proton-Substituted HNaV_6_O_16_ ·4H_2_O Cathode for the Ultrafast-Charging Zinc Storage. ACS Sustain. Chem. Eng..

[31] Bin D., Huo W., Yuan Y., Huang J., Liu Y., Zhang Y., Dong F., Wang Y., Xia Y. (2020). Organic-Inorganic-Induced Polymer Intercalation into Layered Composites for Aqueous Zinc-Ion Battery. Chem.

[32] Chen X., Wang P., Feng Z., Meng C., Zhang Y. (2022). Conductive Polymer Intercalated Vanadium Oxide on Carbon Cloth for Fast Ammonium-Ion Storage in Supercapacitor Applications. Chem. Eng. J..

[33] Li S., Wei X., Wu C., Zhang B., Wu S., Lin Z. (2021). Constructing Three-Dimensional Structured V_2_O_5_/Conductive Polymer Composite with Fast Ion/Electron Transfer Kinetics for Aqueous Zinc-Ion Battery. ACS Appl. Energy Mater..

[34] Hu T., Feng Z., Zhang Y., Liu Y., Sun J., Zheng J., Jiang H., Wang P., Dong X., Meng C. (2021). “Double Guarantee Mechanism” of Ca^2+^-Intercalation and rGO-Integration Ensures Hydrated Vanadium Oxide with High Performance for Aqueous Zinc-Ion Batteries. Inorg. Chem. Front..

[35] Tourneur J., Fabre B., Loget G., Vacher A., Mériadec C., Ababou-Girard S., Gouttefangeas F., Joanny L., Cadot E., Haouas M. (2019). Molecular and Material Engineering of Photocathodes Derivatized with Polyoxometalate-Supported {Mo_3_S_4_} HER Catalysts. J. Am. Chem. Soc..

[36] Ding J., Zheng H., Gao H., Liu Q., Hu Z., Han L., Wang S., Wu S., Fang S., Chou S. (2021). In Situ Lattice Tunnel Distortion of Vanadium Trioxide for Enhancing Zinc Ion Storage. Adv. Energy Mater..

[37] Yang T., Xin D., Zhang N., Li J., Zhang X., Dang L., Li Q., Sun J., He X., Jiang R. (2024). Interfacial Polymerization of PEDOT Sheath on V_2_O_5_ Nanowires for Stable Aqueous Zinc Ion Storage. J. Mater. Chem. A.

[38] Liu Y., Wang T., Sun Y., Zhang M., Gao G., Yang J., Cai K. (2024). Fast and Efficient In-Situ Construction of Low Crystalline PEDOT-Intercalated V_2_O_5_ Nanosheets for High-Performance Zinc-Ion Battery. Chem. Eng. J..

[39] Liu Y., Zhang Y., Jiang H., Sun J., Feng Z., Hu T., Meng C., Pan Z. (2022). Synergistic Engineering of Oxygen-Defect and Heterojunction Boosts Zn^2+^ (De)Intercalation Kinetics in Vanadium Oxide for High-Performance Zinc-Ion Batteries. Chem. Eng. J..

[40] Liao M., Wang J., Ye L., Sun H., Wen Y., Wang C., Sun X., Wang B., Peng H. (2020). A Deep-Cycle Aqueous Zinc-Ion Battery Containing an Oxygen-Deficient Vanadium Oxide Cathode. Angew. Chem. Int. Ed..

[41] Yu S.-B., Lyu H., Tian J., Wang H., Zhang D.-W., Liu Y., Li Z.-T. (2016). A Polycationic Covalent Organic Framework: A Robust Adsorbent for Anionic Dye Pollutants. Polym. Chem..

[42] Pagot G., Benedet M., Maccato C., Barreca D., Di Noto V. (2023). XPS Study of NiO Thin Films Obtained by Chemical Vapor Deposition. Surf. Sci. Spectra.

[43] Mitraka E., Jafari M.J., Vagin M., Liu X., Fahlman M., Ederth T., Berggren M., Jonsson M.P., Crispin X. (2017). Oxygen-Induced Doping on Reduced PEDOT. J. Mater. Chem. A.

[44] Idriss H. (2021). On the Wrong Assignment of the XPS O 1s Signal at 531–532 eV Attributed to Oxygen Vacancies in Photo- and Electro-Catalysts for Water Splitting and Other Materials Applications. Surf. Sci..

[45] Sun J., Rong M., Gao Z., Feng Z., Liu Y., Hu T., Meng C., Zhang Y. (2023). Poly(3,4-Ethylenedioxythiophene) Encapsulating Hydrated Vanadium Oxide Nanobelts Boosts Their Conductivity and Zinc-Ion Storage Properties. Inorg. Chem. Front..

[46] Dai J., Yang C., Xu Y., Wang X., Yang S., Li D., Luo L., Xia L., Li J., Qi X. (2023). MoS2 @Polyaniline for Aqueous Ammonium-Ion Supercapacitors. Adv. Mater..

[47] Sun Y., Huang C., Liu Y., Zhao X., Cai K. (2024). Poly(3,4-Ethylenedioxythiophene)-Coated Vanadium-Doped MnO_2_ Nanorods for High-Performance Flexible Aqueous Zinc-Ion Battery Cathode. ACS Appl. Mater. Interfaces.

[48] Zhao D., Wang X., Zhang W., Zhang Y., Lei Y., Huang X., Zhu Q., Liu J. (2023). Unlocking the Capacity of Vanadium Oxide by Atomically Thin Graphene-Analogous V_2_O_5_·nH_2_O in Aqueous Zinc-Ion Batteries. Adv. Funct. Mater..

[49] Dai Y., Zhang C., Zhang X., Jiang P., Chen J., Zong W., Zheng S., Gao X., Macdonald T.J., He G. (2025). Interfacial Energy Storage in Aqueous Zinc-Ion Batteries. Energy Environ. Sci..

[50] Zhu Y., Wang Y., Li T., Cao K., Hu Y., Pan B., Chen C. (2025). Electrostatic Supramolecular Self-Assembly of Vanadium Oxide and Conductive Polymer for Highly Efficient Zinc Ion Storage. Chem. Eng. J..

[51] Zhu K., Wu T., Huang K. (2019). NaCa_0.6_V_6_O_16_·3H_2_O as an Ultra-Stable Cathode for Zn-Ion Batteries: The Roles of Pre-Inserted Dual-Cations and Structural Water in V_3_O_8_ Layer. Adv. Energy Mater..

[52] Quintanilla-Serrano E.A., Acevedo-Peña P., Díaz-Góngora J.A.I., Borja-Urby R., Reguera E. (2026). Enhancing Electrochemical Zinc-Ion Storage by Exfoliation and rGO Coupling to Dimethylformamide Pre-Intercalated Vanadium (IV) Oxides. J. Mater. Sci..

[53] Ran K., Chen Q., Song F. (2025). Dual-Ion-Modulated Vanadium Oxide Cathode for High-Performance Aqueous Zinc-Ion Batteries. ACS Appl. Mater. Interfaces.

[54] Liu Y., Pan Z., Tian D., Hu T., Jiang H., Yang J., Sun J., Zheng J., Meng C., Zhang Y. (2020). Employing “One for Two” Strategy to Design Polyaniline-Intercalated Hydrated Vanadium Oxide with Expanded Interlayer Spacing for High-Performance Aqueous Zinc-Ion Batteries. Chem. Eng. J..

[55] Hu P., Zhu T., Wang X., Wei X., Yan M., Li J., Luo W., Yang W., Zhang W., Zhou L. (2018). Highly Durable Na_2_V_6_O_16_·1.63H_2_O Nanowire Cathode for Aqueous Zinc-Ion Battery. Nano Lett..

[56] Naikwade M.B., Katkar P.K., Lee S.-W. (2024). Understanding the Impact of Porosity on Li-Ion Diffusion Enhancement in Micro-Sized Silicon Particles for Advanced Batteries. Ceram. Int..

[57] Naikwade M.B., Katkar P.K., Lee S.-W. (2025). Superior Performance of an Ultrathin Pyridinic-Layered Micro-Structural Porous Silicon Anode with a Silicon Content Exceeding 99%. J. Mater. Chem. A.

[58] Wu M., Shi C., Yang J., Zong Y., Chen Y., Ren Z., Zhao Y., Li Z., Zhang W., Wang L. (2024). The LiV_3_O_8_ Superlattice Cathode with Optimized Zinc Ion Insertion Chemistry for High Mass-Loading Aqueous Zinc-Ion Batteries. Adv. Mater..

[59] Wang S., Yao S., Dai N., Fu W., Liu Y., Ji K., Ji Y., Yang J., Liu R., Li X. (2024). Spin Symmetry Breaking-Induced Hubbard Gap Near-Closure in N-Coordinated MnO_2_ for Enhanced Aqueous Zinc-Ion Battery Performance. Angew. Chem. Int. Ed..

[60] Lin Y., Meng J., Hei P., Wang Y., Li B., Sun X., Song Y., Liu X. (2025). Iodine-Mediated Defect Engineering of Vanadyl Phosphate Cathodes for High-Performance Aqueous Zinc-Ion Batteries. Adv. Funct. Mater..

[61] Zhu Y., Zeng S., Deng W., Si J., Pan B., Chen C. (2024). Heterovalent Dual-Ion Interlayer-Confined Vanadium Oxide Nanobelts as a Stable Cathode for Zinc Storage. J. Energy Storage.

