# Supplementary material for: PEDOT-Regulated Interfacial Engineering of Sodium Vanadium Oxide Nanostructures for High-Performance Aqueous Zinc-Ion Batteries
Source: Nanomaterials (Basel). 2026 Jun 12;16(12):729. doi: 10.3390/nano16120729 (PMC13305433; doi:10.3390/nano16120729)
Supplement: Supplementary file 1 [file nanomaterials-16-00729-s001.zip › nanomaterials-4347044-supplementary.pdf]

PEDOT-Regulated Interfacial Engineering of Sodium Vanadium Oxide Nanostructures for High-Performance Aqueous Zinc-Ion Batteries

Zeeshan Umar[1,2], Jiangfeng Gong*[1,2], Guangchao Du[1], Wenyi He[1], Chunmei Tang[2], Jingjing Xu[3], Yuwu Cai[3], Xinyi Zhao[3]

1. State Key Laboratory of Vanadium and Titanium Resources Comprehensive Utilization, Panzhihua 617000, China
2. College of Mechanics and Engineering Science, Hohai University, Nanjing 210098, China
3. Jiangsu Provincial Institute of Product Quality Supervision and Inspection, Nanjing 210007, China

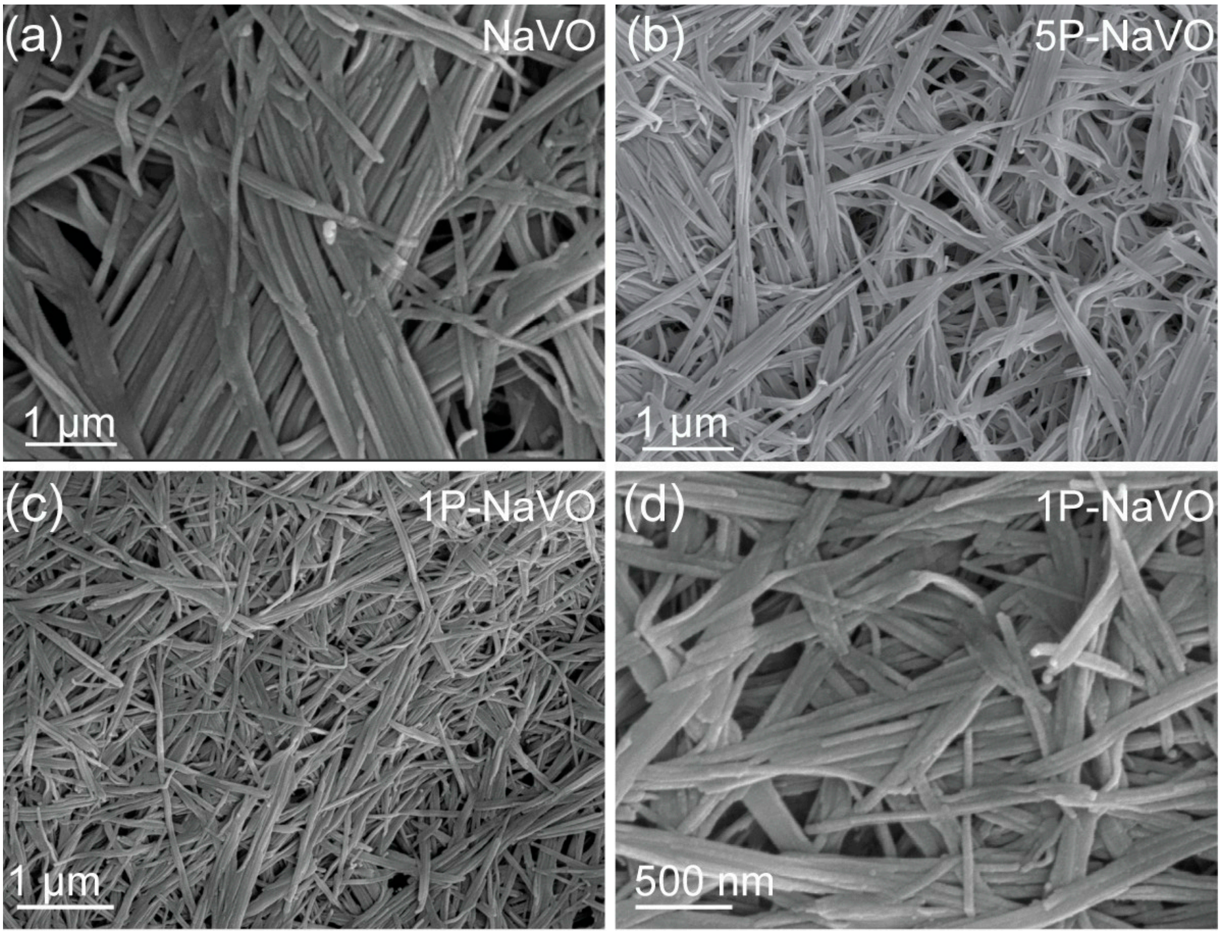

Figure S1. (a) SEM image of NaVO; (b) SEM image of 5P-NaVO; (c-d) SEM images of 1P-NaVO.

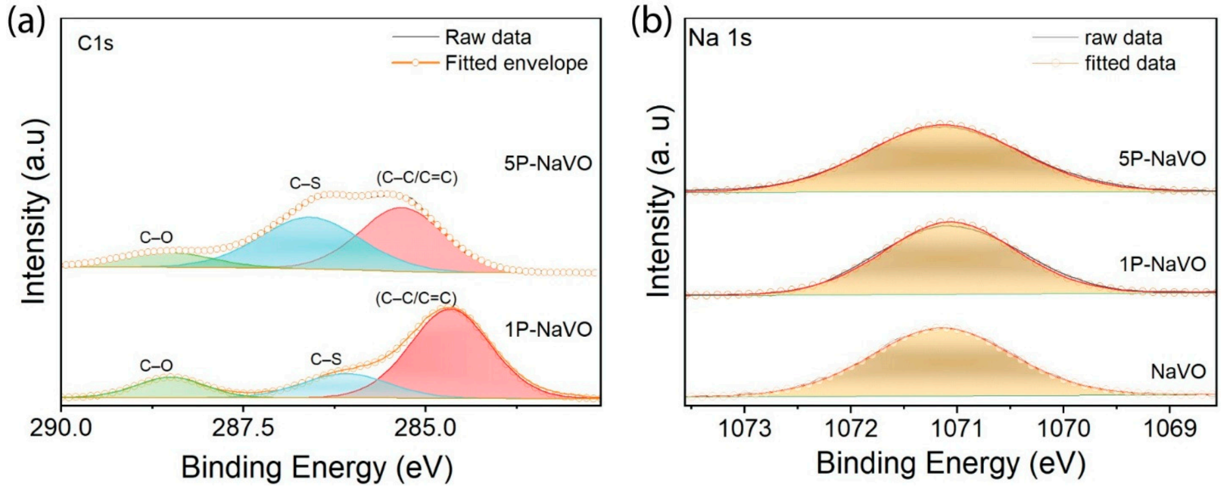

Figure S2. High-resolution XPS spectra of (a) C 1s; (b) Na 1s.

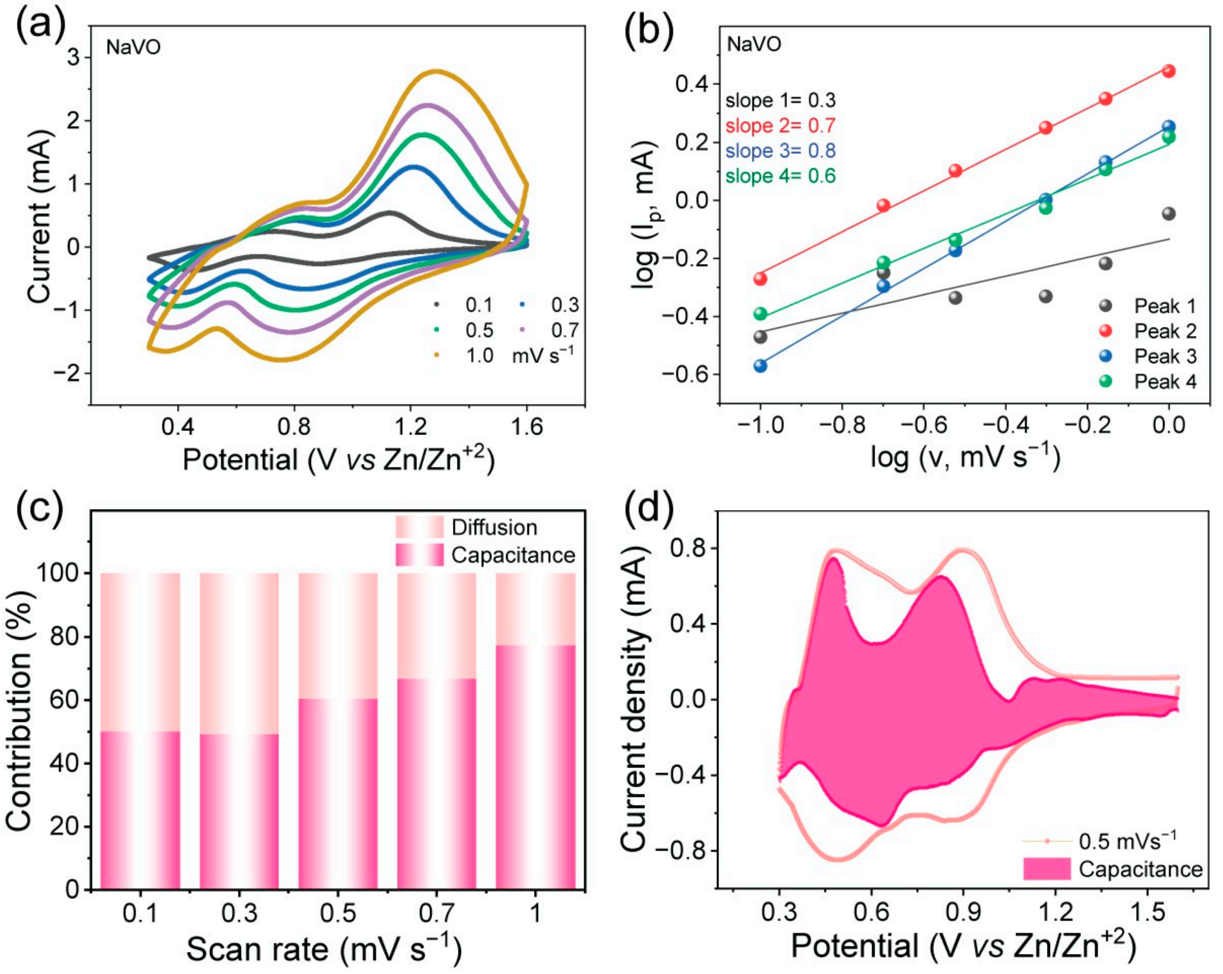

Figure S3. (a) CV curves of NaVO at different scan rates from 0.1 to 1.0 mV s^{-1}. (b) Log(i) versus log(v) plots for calculating b values of the selected redox peaks. (c) Capacitive and diffusion-controlled contribution ratios at different scan rates. (d) Capacitive contribution at 0.5 mV s^{-1}.

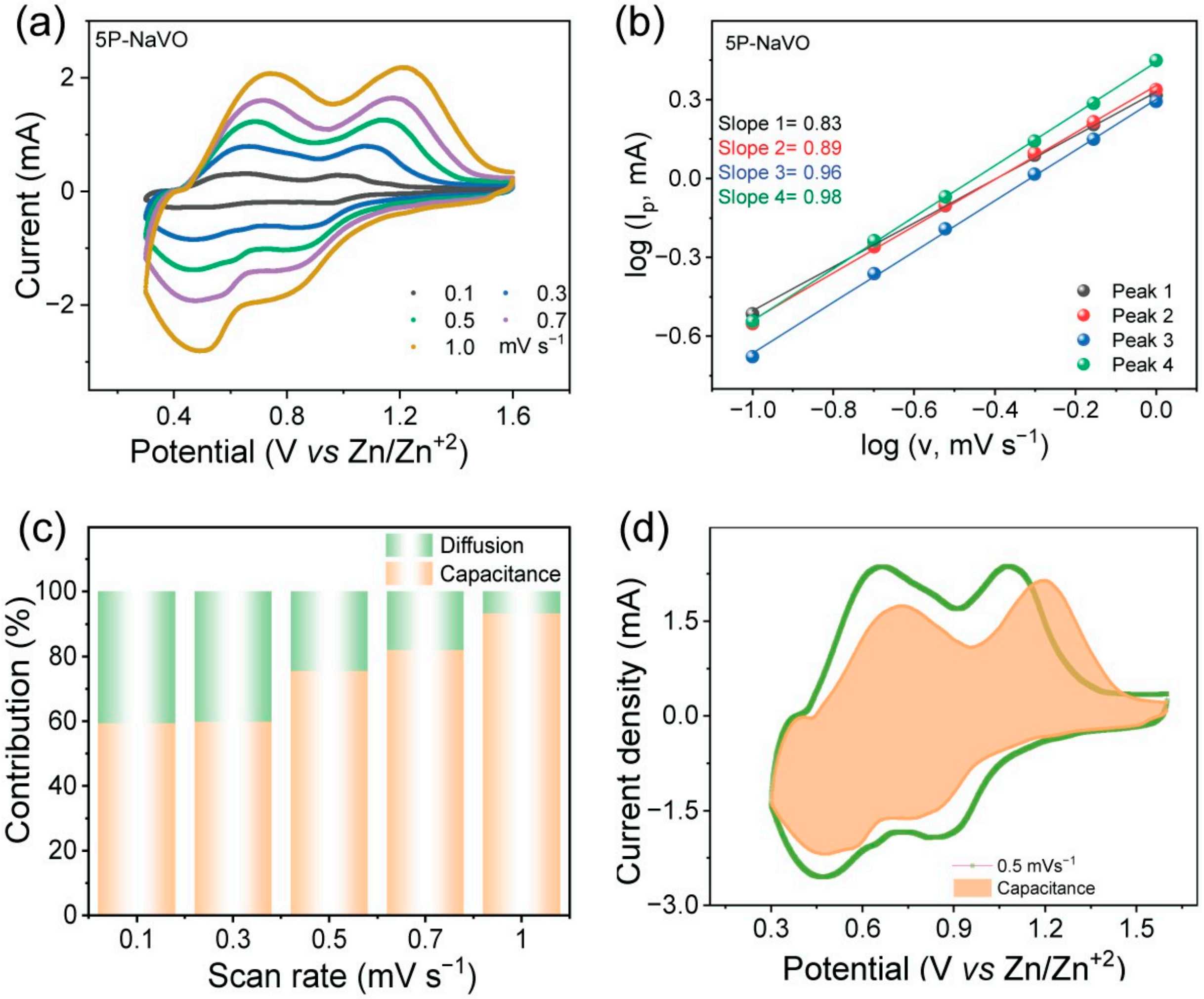

Figure S4. (a) CV curves of 5P-NaVO at different scan rates from 0.1 to 1.0 mV s^{-1}. (b) Log(i) versus log(v) plots for calculating b values of the selected redox peaks. (c) Capacitive and diffusion-controlled contribution ratios at different scan rates. (d) Capacitive contribution at 0.5 mV s^{-1}.

Note 1

Temperature-Dependent EIS Fitting and Activation Energy Calculation

Temperature-dependent EIS analysis was performed to examine the interfacial charge-transfer behavior of the three prepared cathodes. The measurements were carried out under open-circuit voltage conditions from 20 to 50 °C. All EIS spectra were fitted using ZView2 software with the same equivalent circuit, Rs–(Rct||CPE1), as shown in Figure 5e. In this circuit, Rs denotes the overall ohmic resistance of the cell, Rct represents the interfacial charge-transfer resistance, and CPE1 accounts for the non-ideal capacitive response of the electrode surface. Although the fitted values changed with the electrode material and testing temperature, the circuit model was kept consistent for all samples.

The charge-transfer activation energy was obtained from the temperature-dependent Rct values using the Arrhenius relationship:

$$\frac{1}{R_{ct}} = A_0 e^{-\frac{E_{act}}{k_B T}} \qquad (eq.\ S1)$$

where A_0 is a pre-exponential constant, E_{act} is the activation energy, k_B is the Boltzmann constant (k_B =1.380649×10^{-23} J K^{-1}), and T is the absolute temperature (K).

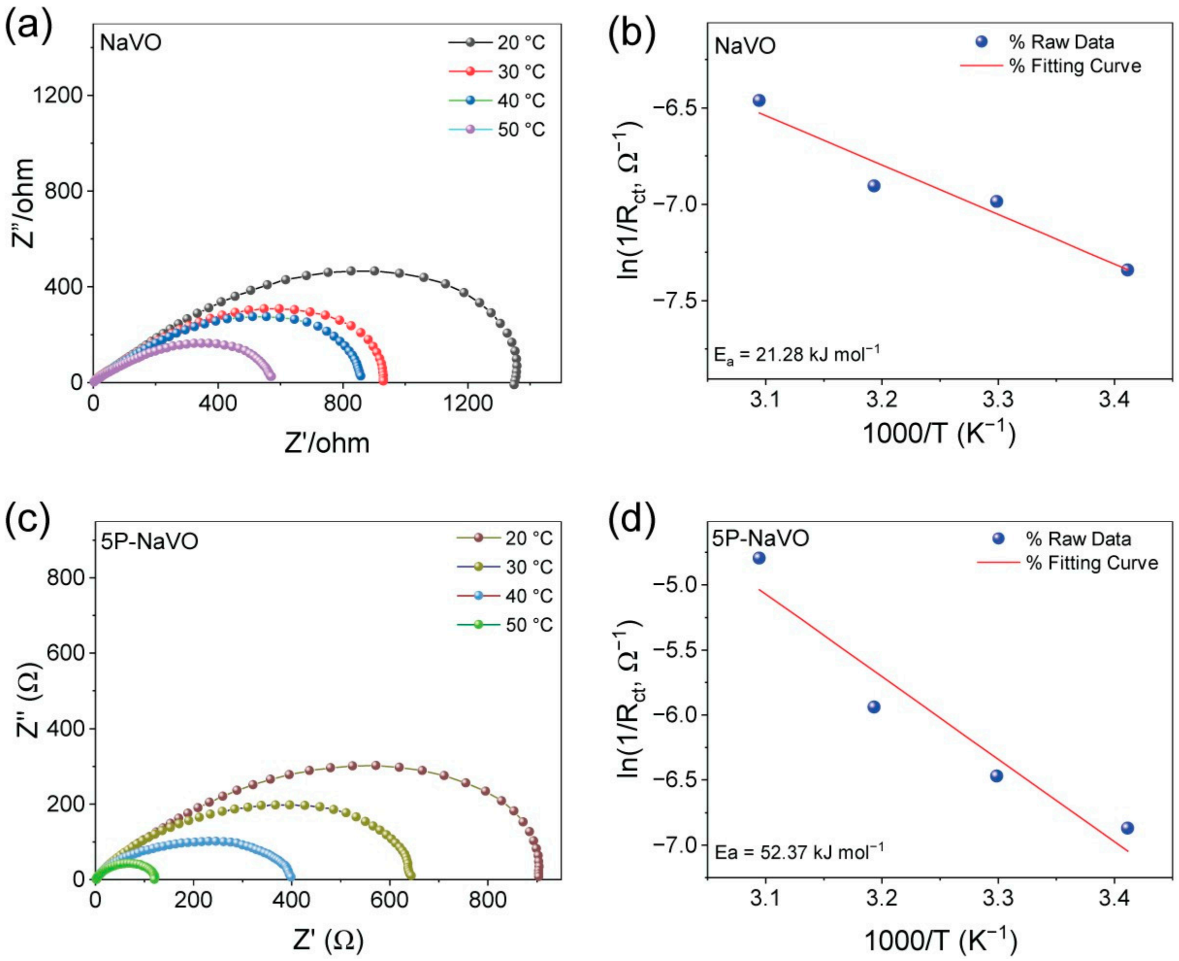

Figure S5. (a) Nyquist plots of NaVO measured at different temperatures. (b) Arrhenius fitting plot for calculating the activation energy of NaVO. (c) Nyquist plots of 5P-NaVO measured at different temperatures. (d) Arrhenius fitting plot for calculating the activation energy of 5P-NaVO.

Note 2:

Calculation of Zn^{2+} Diffusion Coefficients from GITT Measurements

To investigate the Zn^{2+} transport kinetics within the electrode materials, galvanostatic intermittent titration technique (GITT) measurements were performed within a voltage window of 0.3 to 1.6 V. During the test, the cells were alternately subjected to galvanostatic charging and discharging at a current density of 0.1 A g^{-1} for 10 min and then allowed to rest under open circuit conditions for 30 min, enabling the cell potential to gradually reach a near equilibrium state. The Zn^{2+} diffusion coefficient ($D_{Zn^{2+}}$) was subsequently determined from the obtained GITT curves according to the following expression:

$$D_{Zn^{2+}} = \frac{4}{\pi\tau}\left(\frac{n_m . V_m}{S}\right)^2 . \left(\frac{\Delta E_s}{\Delta E_\tau}\right)^2$$

In this equation, τ denotes the applied current-pulse duration, n_m corresponds to the molar quantity of the electrochemically active material, V_m refers to its molar volume, and S represents the effective interfacial area between the electrode and electrolyte. ΔE_s is the equilibrium potential difference measured between two successive relaxation states, and ΔE_τ indicates the potential variation recorded during the current pulse.

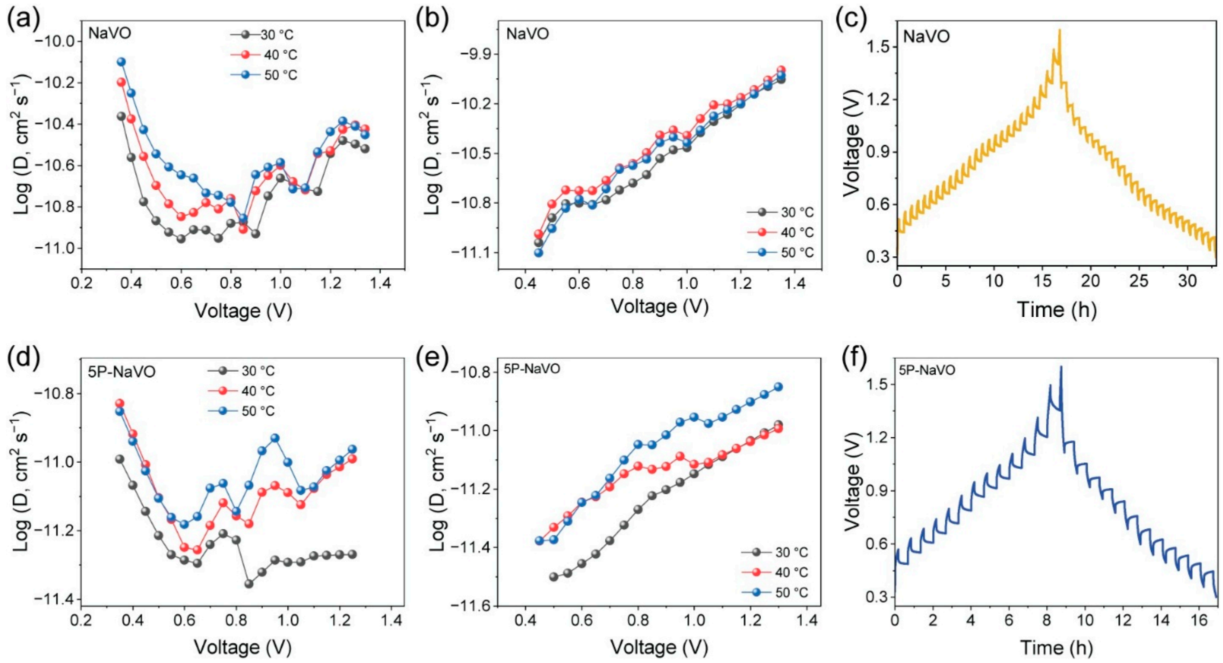

Figure S6. (a,b) Zn^{2+} diffusion coefficients of NaVO during charge and discharge at different temperatures. (c) GITT voltage profile of NaVO. (d,e) Zn^{2+} diffusion coefficients of 5P-NaVO during charge and discharge at different temperatures. (f) GITT voltage profile of 5P-NaVO.

Table S1: Reported D_{Zn}^{2+} values of the vanadium-based cathodes.

**Material**	**Battery system**	**D_{Zn} value**	**Method**	**Reference**
NaVO	AZIB cathode	10^{-11} to 10^{-10} $cm^2\ s^{-1}$	GITT	This work
1P-NaVO	AZIB cathode	$10^{-10.8}$ to $10^{-9.8}$ $cm^2\ s^{-1}$	GITT	This work
5P-NaVO	AZIB cathode	$10^{-11.5}$ to $10^{-10.8}$ $cm^2\ s^{-1}$	GITT	This work
PEO-LVO	AZIB cathode	Close to $10^{-10.8}$ $cm^2\ s^{-1}$	GITT	[1]
TEAMO	AZIB cathode	10^{-12} to 10^{-10} $cm^2\ s^{-1}$	GITT	[2]
VOP-I4	AZIB cathode	10^{-14} to 10^{-13} $cm^2\ s^{-1}$	GITT	[3]
KCVO	AZIB cathode	8.62×10^{-10} $cm^2\ s^{-1}$	GITT	[4]

References

1. Wu, M.; Shi, C.; Yang, J.; Zong, Y.; Chen, Y.; Ren, Z.; Zhao, Y.; Li, Z.; Zhang, W.; Wang, L.; et al. The $LiV_3 O_8$ Superlattice Cathode with Optimized Zinc Ion Insertion Chemistry for High Mass-Loading Aqueous Zinc-Ion Batteries. *Advanced Materials* **2024**, *36*, 2310434, doi:10.1002/adma.202310434.
2. Wang, S.; Yao, S.; Dai, N.; Fu, W.; Liu, Y.; Ji, K.; Ji, Y.; Yang, J.; Liu, R.; Li, X.; et al. Spin Symmetry Breaking-Induced Hubbard Gap Near-Closure in N-Coordinated MnO_2 for Enhanced Aqueous Zinc-Ion Battery Performance. *Angew Chem Int Ed* **2024**, *63*, e202408414, doi:10.1002/anie.202408414.
3. Lin, Y.; Meng, J.; Hei, P.; Wang, Y.; Li, B.; Sun, X.; Song, Y.; Liu, X. Iodine-Mediated Defect Engineering of Vanadyl Phosphate Cathodes for High-Performance Aqueous Zinc-Ion Batteries. *Adv Funct Materials* **2025**, *35*, 2415639, doi:10.1002/adfm.202415639.
4. Zhu, Y.; Zeng, S.; Deng, W.; Si, J.; Pan, B.; Chen, C. Heterovalent Dual-Ion Interlayer-Confined Vanadium Oxide Nanobelts as a Stable Cathode for Zinc Storage. *Journal of Energy Storage* **2024**, *97*, 112836, doi:10.1016/j.est.2024.112836.
